# High co-expression of the SDF1/CXCR4 axis in hepatocarcinoma cells is regulated by AnnexinA7 in vitro and in vivo

**DOI:** 10.1186/s12964-018-0234-1

**Published:** 2018-05-21

**Authors:** Jingwen Wang, Yuhong Huang, Jun Zhang, Boyi Xing, Wei Xuan, Honghai Wang, He Huang, Jiayu Yang, Jianwu Tang

**Affiliations:** Department of Pathology, Dalian Medical University, Key Laboratory for Tumor Metastasis and Intervention of Liaoning Province, 9 West, Lvshun Southern Road, Dalian, 116044 Liaoning China

**Keywords:** SDF1, CXCR4, AnnexinA7, VEGFC/D-VEGFR3/NRP2, Hepatocarcinoma

## Abstract

**Background:**

SDF1/CXCR4 and AnnexinA7 play important roles in many physiological and pathological conditions, but the molecular association between them in cancer cells has not been studied thus far.

**Methods:**

The expression changes of SDF1/CXCR4 were detected by gene transcriptome sequencing, qRT-PCR, Western blotting, cytoimmunofluorescence and immunohistochemistry in mouse hepatocarcinoma F/P cells, AnnexinA7 downregulated expression F (F_A7DOWN_) cells, AnnexinA7 overexpression P (P_A7UP_) cells, AnnexinA7 unrelated sequence F (F_SHUS_) cells, empty vector P (P_NCEV_) cells and normal liver cells in vitro and in vivo*.*

**Results:**

SDF1 and CXCR4 were co-expressed in hepatocarcinoma cells. SDF1 was localized mainly in the cytoplasm of cells, while CXCR4 was mainly localized in the cell membrane. Both in vitro and in vivo*,* expression levels of SDF1/CXCR4 in F and P cells were higher than in normal liver cells, and expression levels of SDF1/CXCR4 in F cells with high lymphatic metastatic potential were higher than those in P cells with low lymphatic metastatic potential*.* Expression of SDF1 was higher than that of CXCR4 in P cells and normal liver cells, while expression of CXCR4 was higher than that of SDF1 in F cells. Expression levels of SDF1/CXCR4 were completely consistent with AnnexinA7 regulation. After the AnnexinA7 gene was downregulated or upregulated, expression levels of SDF1/CXCR4 in F_A7DOWN_/P_A7UP_ cells were lower or higher than those in F_SHUS_/P_NCEV_ cells*.* Furthermore, CXCR4 was more sensitively modulated by AnnexinA7 regulation than SDF1.

**Conclusions:**

High co-expression of SDF1/CXCR4 is a molecular characteristic of hepatocarcinoma cells, especially those with high lymphatic metastatic potential. AnnexinA7 positively regulates expression levels of SDF1/CXCR4, in particular CXCR4, and AnnexinA7 is a functional regulator of SDF1/CXCR4*.*

## Background

The ligand stromal cell-derived factor 1 (SDF1) and its receptor, chemokine Cys-X-Cys receptor 4 (CXCR4), have received great attention in recent years. As an active biomolecular axis, SDF1/CXCR4 participates in a variety of physiological and pathological conditions, including hematopoiesis, embryonic development, cell chemotaxis, cell calcium influx, immune tolerance and inflammation as well as tumor proliferation and migration. Some studies have revealed that SDF1 and CXCR4 are not only expressed in macrophages, hematopoietic cells, vascular endothelial cells, muscle cells, heart tissue, liver tissue, kidney tissue, brain tissue and skin tissues but are also expressed in many types of tumor cells [1, 2]. Our previous studies found that AnnexinA7 is an important differential gene that is closely related to the biological behaviors of cell membrane transport, signal transduction, proliferation and invasion of tumor cells [3–5]. However, the molecular association of AnnexinA7 and the SDF1/CXCR4 axis has not been studied thus far. This study focused on the expression characteristics of the SDF1 ligand and CXCR4 receptor before or after AnnexinA7 downregulation and upregulation in mouse hepatocarcinoma cells, which has important clinical significance to clarify the molecular mechanism of tumor development and progression.

## Methods

### Cell cultures and lentiviral transfection in vitro

The mouse hepatocarcinoma cell lines HcaF and HcaP, with lymph node metastatic rates greater than 70% and less than 30%, respectively, were established and maintained by our laboratory [6]. The mouse normal liver cell line NCTC 1469, was purchased from Cell Tech Company. HcaF/P (abbreviated as F/P) cells and normal NCTC 1469 (abbreviated as normal) cells were cultured in 90% RPMI 1640 (Gibco, USA) supplemented with 15% fetal bovine serum (FBS; PAA, USA) and 100 U/ml penicillin (Sigma, USA). Cells were seeded into a 6-well plate (200 ml/well), and 30 μl of virus solution containing LV-AnnexinA7-shRNA, LV-AnnexinA7-overexpression, LV-AnnexinA7-Fsh-unrelated sequence, or LV-AnnexinA7-NC-empty vector was added to the cultured F/P cells. Lentiviral vector information (Gene Chem, China) is listed in Table 1. After 10 days of screening with 400 mg/ml G418 (Invitrogen, USA), nearly 90% of cells survived. Through the above procedures, the stable AnnexinA7 downregulated F (F_A7DOWN_) cells, AnnexinA7 overexpression P (P_A7UP_) cells, AnnexinA7 unrelated sequence F (F_SHUS_) cells, and empty vector P (P_NCEV_) cells (abbreviated as cells in vitro) were established.

**Table 1:** Vector information for AnnexinA7 silencing and overexpression

Vector Name	The elements order of vector	Resistance	Fluorescent tags
GV248 (Silence Vector)	Hu6-MSC-Ubiquitin-EGFP-IRES-puromycin	Puromycin	EGFP
GV492 (Over-expressions Vector)	Ubi-MCS-3FLAG-CBh-gcGFP-IRES-puromycin	Puromycin	gcGFP

### Transplanted tumor models in vivo

The experimental animals were inbred Chinese 615 mice (6–8 weeks old and 18–22 g of weight) provided by the Experimental Animal Center of Dalian Medical University. A total of 48 inbred Chinese 615 mice were divided into 6 groups with 8 mice in each group, and normal liver tissue of 615 mice was used as the control. F_A7DOWN_/P_A7UP_, F_SHUS_/P_NCEV_ and F/P cells (1 × 10^6^/0.05 ml) were subcutaneously inoculated into the foot pad of 615 mice to establish corresponding transplanted tumor models (abbreviated as cells in vivo). On the 28th day of post inoculation, mice were sacrificed, and samples from tumor tissues were prepared for qRT-PCR, Western blotting, hematoxylin-eosin staining and immunohistochemistry staining.

### cDNA library preparation and transcriptome sequencing in vitro

According to the manufacturer’s instructions, total RNA was extracted from fresh F/P, F_A7DOWN_/F_SHUS,_ and P_A7UP_/P_NCEV_ cells in vitro. After validating the integrity and purity of RNA, a cDNA library was constructed based on the standard NEB next Ultra RNA library Prep Kit (Invitrogen). Short double-stranded cDNA fragments were purified. DNA ends were repaired, and ‘A’ bases were added. After screening approximately 200 bp cDNA with AMPure XP beads, PCR amplification and product purification were performed. The cDNA library was obtained and sequenced through Illumina HiSeq PE125/PE150 to obtain differential expressed genes (DEGs) between experiment and control groups. The criterion was *P* < 0.05, and the results were analyzed with DESeq2 software (Novogene Company, China).

### Real-tixe-PCR analysis in vitro and in vivo

For qRT-PCR analysis of SDF1 and CXCR4 mRNA expression, total RNA from F_A7DOWN_/P_A7UP_, F_SHUS_/P_NCEV_ and original F/P cells as well as normal liver cells in vitro and in vivo were extracted using Trizol reagent (Invitrogen, USA). Reverse transcription of purified RNA was performed using the PrimeScript1 RT reagent kit (Takara, Japan). Quantification of gene transcripts was performed by qRT-PCR (Fluorescence real-time quantitative PCR meter MX3005P, USA) using SYBR1 Premix Ex TaqTM II (Takara, Japan), and the levels were normalized to GAPDH as the internal control. Primer sequences (Takara, Japan) for SDF1, CXCR4 and GAPDH are listed in Table 2. MXP software was used to analyze the results. Differences in mRNA expression were calculated according to the^△△Ct^ method and displayed as 2(^—△△Ct^).

**Table 2:** Primer sequences for SDF1, CXCR4 and GAPDH

Sequence	Forward primer (5′ → 3′)	Reverse (5′ → 3′)
Gene name		
SDF1	CCTGTGTGTCATGCCCTCTT	AGTCCAGCCTGCTATCCTCA
CXCR4	GTCAACCTCTAGAGCAGCGT	CTATCGGGGTAAAGGCGGTC
GAPDH	AAATGGTGAAGGTCGGTGTGAAC	CAACAATCTCCACTTTGCCACTG

### Protein extraction and western blot analysis in vitro and in vivo

The six groups of cells, namely, F_A7DOWN_/P_A7UP_, F_SHUS_/P_NCEV_, F/P and normal liver cells in vitro and in vivo, were collected. The concentration of total proteins was determined by a BCA protein kit (Thermo Fisher Scientific, USA). Total protein (50 μg) from each group was loaded and separated by 12% sodium dodecyl sulfate-polyacrylamide gel electrophoresis (SDS-PAGE) and transferred to polyvinylidene fluoride (PVDF) membranes (Millipore, USA). Membranes were then incubated with antibodies against AnnexinA7 (Abcam, USA, 1:1500), SDF1 (Santa Cruz, CA, 1:400), CXCR4 (Santa Cruz, CA, 1:400), and GAPDH (ZSGB-Bio, China, 1:2000) overnight at 4 °C followed by incubation with fluorescent secondary antibodies (IRDye 800CW donkey anti-mouse/Rabbit; LI-COR, USA 1:16000) for 1.5 h at room temperature. Western blot results were visualized by the Odyssey Infrared Imaging System (LI-COR Biosciences, USA). Protein expression was represented as a value relative to GAPDH expression. ImageJ software was used to calculate IOD values.

### Cytoimmunofluorescence in vitro and immunocytochemistry in vivo

For cytoimmunofluorescence (CIF), F_A7DOWN_/P_A7UP_, F_SHUS_/P_NCEV_, F/P cells and normal liver cells in vitro were incubated with SDF1 (Santa Cruz, CA, 1:75) and CXCR4 (Santa Cruz, CA, 1:75) polyclonal antibodies at 4 °C overnight followed by incubation with fluorescence secondary antibodies (DyLight 594 AffiniPure Donkey Anti-Rat/Mouse; Abbkine, USA, 1:50) at 37 °C for 90 min, and the cell nuclei were stained with 4′, 6-diamidino-2-phenylindole diaminobenzidine (DAPI). Cytoimmunofluorescence was observed under laser confocal microscope, and Image J software was used to calculate average OD values.

Immunohistochemistry (IHC) was used to detect the expression of AnnexinA7, SDF1 and CXCR4 in F_A7DOWN_/P_A7UP_, F_SHUS_/P_NCEV_, F/P cells and normal liver tissue in vivo. A mouse monoclonal antibody against AnnexinA7 and rabbit polyclonal antibodies against SDF1 and CXCR4 were used at dilutions of 1:1500, 1:400 and 1:400, respectively. A Ready-to-use Elivision TM plus Polymer HRP (Mouse/Rabbit) IHC Kit (Fuzhou Mai Xin Biotech, China) was used as the secondary antibody. Protein expression levels were quantified based on the intensity and uniformity of nuclear/cytoplasmic staining, and ImageJ software was used to calculate average OD values.

### Statistical analysis

Statistical analyses were performed using SPSS17.0 software (SPSS company, IN). All data were represented as the mean standard deviation derived from at least three independent experiments. Analysis of variance (ANOVA) or one-way ANOVA for repeated measures coupled with the x^2^-test were performed to determine if there were differences between the in vitro and in vivo *assays*. A *P*-value less than 0.05 was considered statistically significant.

## Results

### Establishment of F_A7DOWN_/P_A7UP_, F_SHUS_/P_NCEV_, _and_ F/P cells in vitro and corresponding transplanted tumor models in vivo

**Fig. 1:**
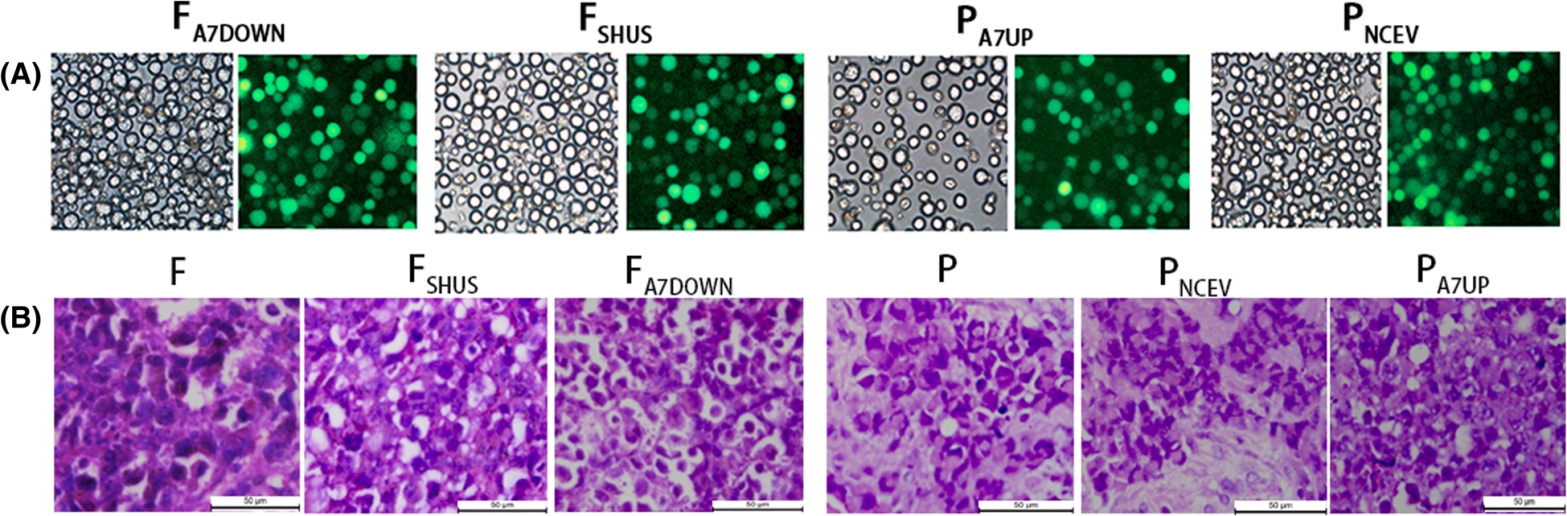
Establishment of F_A7DOWN_/P_A7UP_, F_SHUS_/P_NCEV_, and F/P cells in vitro as well as corresponding transplanted tumor models in vivo. The magnification and green fluorescence in stable transfected F_A7DOWN_, F_SHUS_, P_A7UP_, and P_NCEV_ cells in vitro (A) observed by inverted microscope and fluorescence microscope. 200 x magnification. Hematoxylin-eosin staining of F, F_SHUS_, F_A7DOWN_, P, P_NCEV_, and P_A7UP_ cells in vivo (B) observed by optical microscope. 400 x magnification. (Bar = 50 μm)

**Fig. 2:**
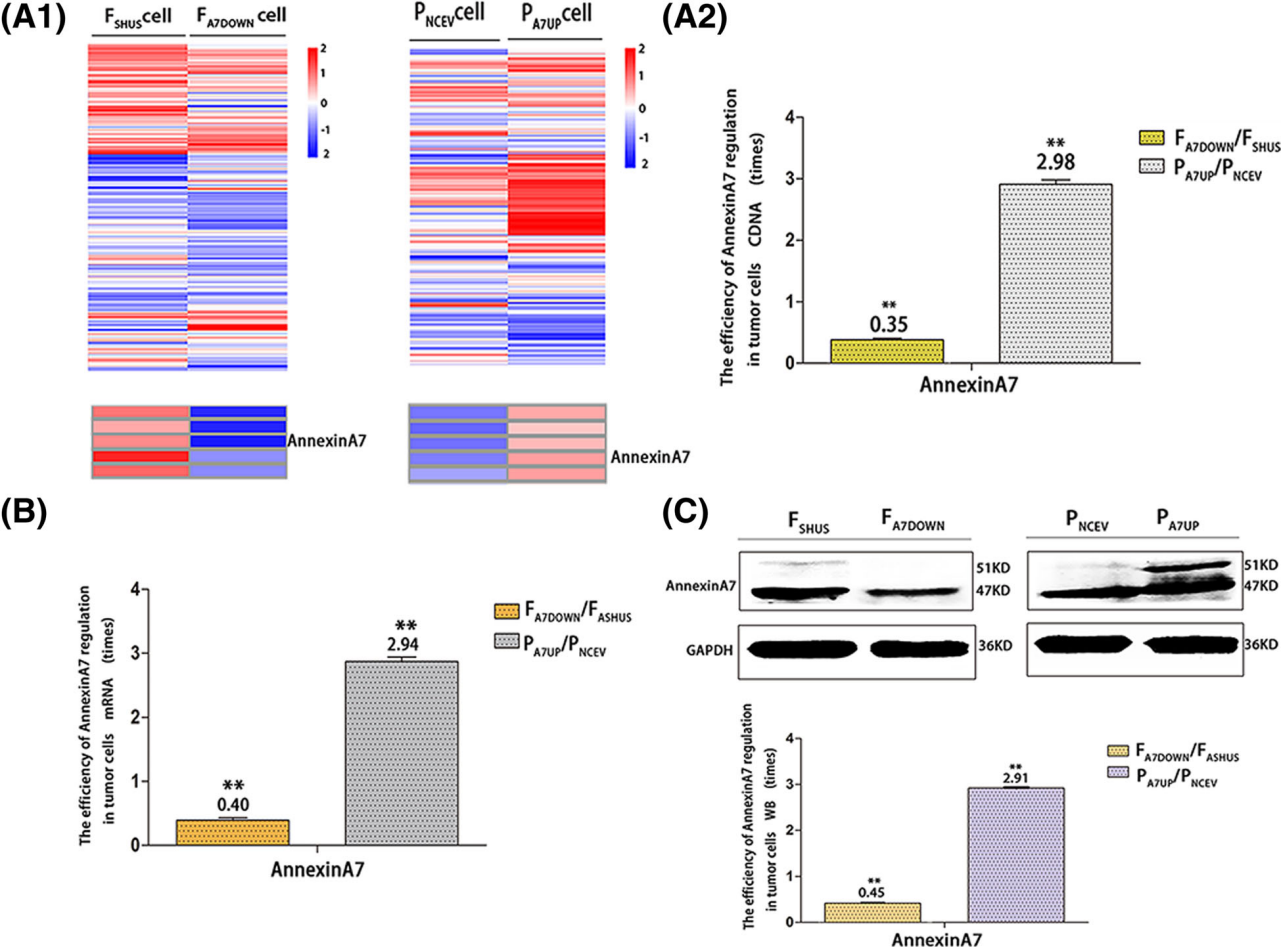
The efficiency of AnnexinA7 regulation in stable transfected F_A7DOWN_/P_A7UP_ and F_SHUS_/P_NCEV_ cells in vitro as analyzed by transcriptome sequencing, qRT-PCR and Western blotting. Transcriptome sequencing heat maps (A1) and cDNA regulation efficiency (A2) of AnnexinA7 in F_A7DOWN_/P_A7UP_ and F_SHUS_/P_NCEV_ cells in vitro*.* mRNA regulation efficiency of AnnexinA7 in vitro (B). Western blot results and protein regulation efficiency of AnnexinA7 in vitro (C)

There are clear green fluorescent tags in the cytoplasm of lentiviral stable transfected F_A7DOWN_/P_A7UP_ and F_SHUS_/P_NCEV_ cells in vitro and in vivo observed by fluorescence microscope. The numbers of cells with green fluorescence exceeded 95% of the total number of cells in each group, while there was no obvious green fluorescence in F/P cells, normal liver cells and normal liver tissue in vitro and in vivo (Fig. 1A). HE, CIF and IHC staining showed that F/P, F_A7DOWN_/P_A7UP_ and F_SHUS_/P_NCEV_ cells in vitro and in vivo all grew and were activated in good condition (Figs. 1B, 4A, C and 6A, B, C, D). Compared to F_SHUS_/P_NCEV_ cells, the efficiency of AnnexinA7 gene downregulation or upregulation in F_A7DOWN_ or P_A7UP_ cells was indicated by cDNA, mRNA and Western blot protein analyses as follows: it decreased by 64.64% (cDNA, in vitro), 59.35% (mRNA, in vitro) and 54.53% (protein, in vitro); or increased by 1.98 times (cDNA, in vitro), 1.94 times (mRNA, in vitro) and 1.91 times (protein, in vitro), (Fig. 2).

### Expressions of SDF1 and CXCR4 in different hepatocarcinoma cells with high/low lymphatic metastatic potentials and normal liver cells in vitro and in vivo

**Fig. 3:**
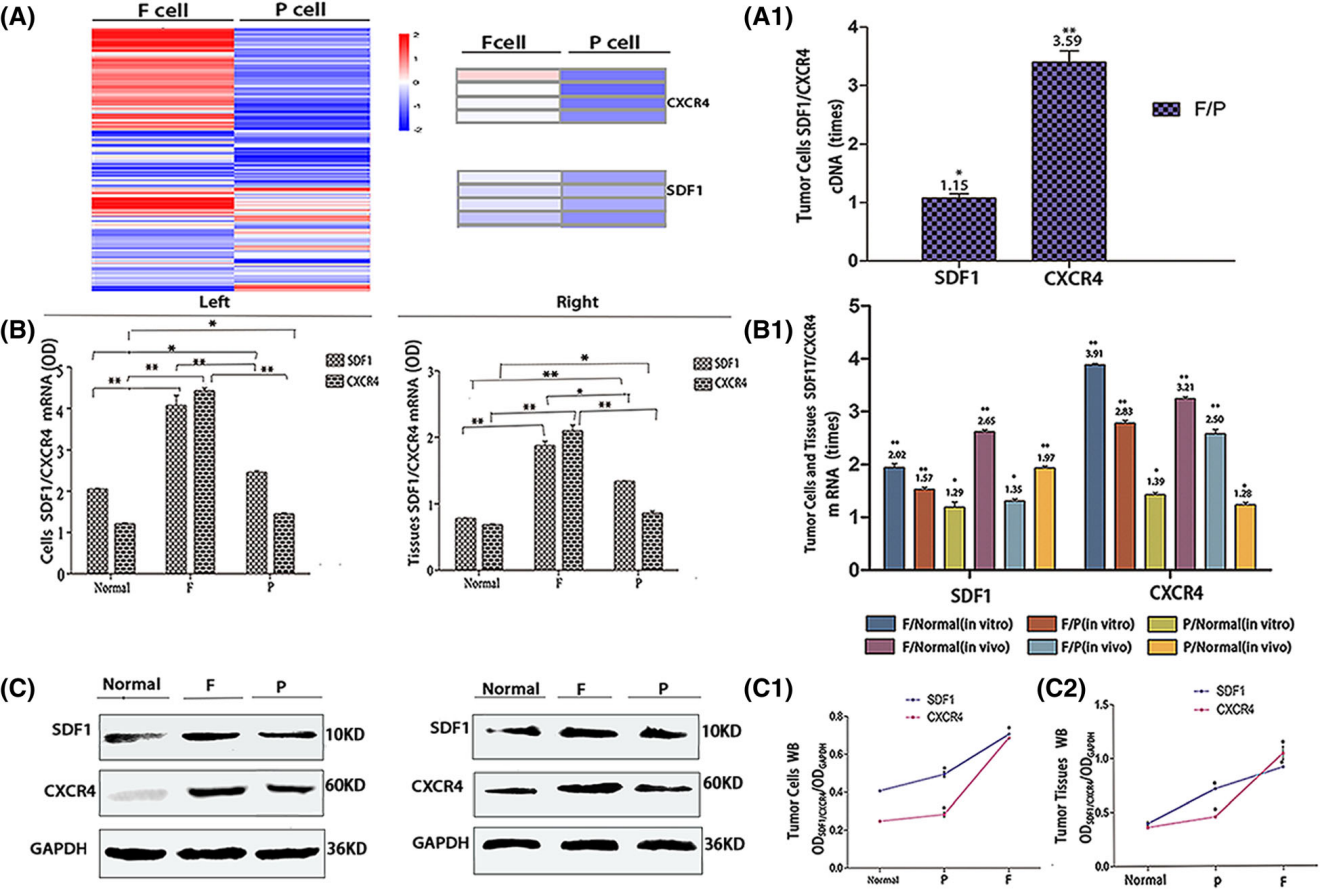
Expression of SDF1 and CXCR4 in different hepatocarcinoma cells and normal liver cells in vitro and in vivo. Transcriptome sequencing heat maps (A) and cDNA expression (A1) of SDF1/CXCR4 in F/P cells in vitro. qRT-PCR (B, B1) and Western blot (C, C1, C2) analysis of SDF1/CXCR4 mRNA and protein expressions in normal hepatocytes and F/P cells in vitro (Left) and in vivo (Right). mRNA comparison between different groups in vitro and in vivo (B1). Protein expression for in vitro (C, Left) and in vivo (C, Right). Western blot OD densities for in vitro (C1) and in vivo (C2)

**Fig. 4:**
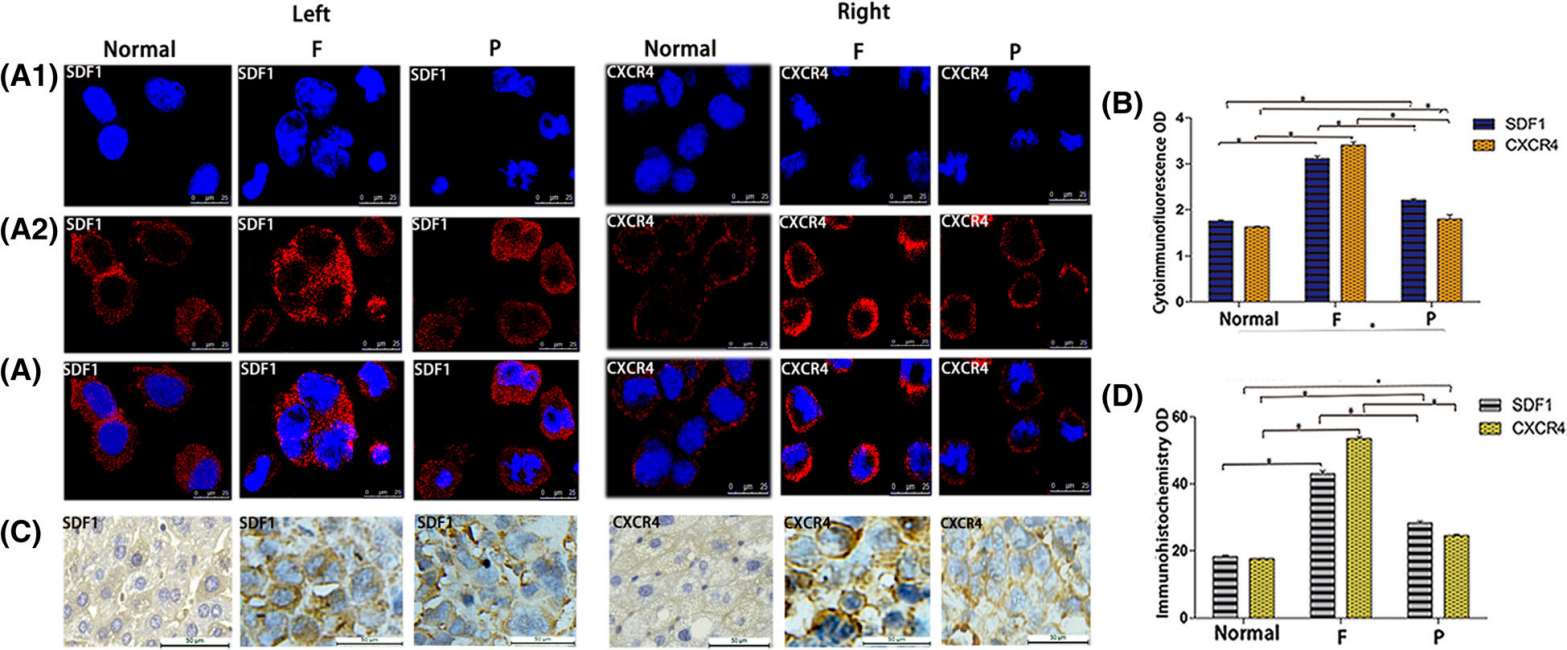
Expression of SDF1 and CXCR4 in different hepatocarcinoma cells and normal liver cells in vitro and in vivo.Cytoimmunofluorescence cell nuclear DAPI staining (A1), cell staining (A2), merged picture(A) and immunohistochemistry (C)] analysis of SDF1 (Left) and CXCR4 (Right) expression in normal hepatocytes, F/P cells in vitro (A1, A2, A) and in vivo (C). SDF1 expression in vitro (A1, A2, A, Left). CXCR4 expression in vitro (A1, A2, A, Right). SDF1 expression in vivo (C, Left). CXCR4 expression in vivo (C, Right). Cytoimmunofluorescence and immunohistochemistry OD values for SDF1/CXCR4 in normal hepatocytes and F/P cells in vitro (B) and in vivo (D). _*_ indicates *P* < 0.05 as statistically significant

Transcriptome sequencing, qRT-PCR, Western blotting, cytoimmunofluorescence and immunohistochemistry confirmed that in the three groups of F/P cells and normal liver cells in vitro and in vivo, SDF1 was mainly localized in the cytoplasm of cells, and in a small amount, it was located in the cell membrane; while CXCR4 was mainly localized in the cell membrane, and in a small amount, it was localized in the cytoplasm (Fig. 4 A1, A2, A, C). The expression levels of SDF1/CXCR4 were all higher in F and P tumor cells than in normal liver cells and tissues in vitro and in vivo. The SDF1 levels in F/P cells were 2.65 times (mRNA, in vivo) and 1.91 times (protein, in vivo) higher than those of normal liver tissues (*P* < 0.05), and the CXCR4 levels in F/P cells were 3.91 times (mRNA, in vitro) and 1.39 times (mRNA, in vitro) higher than those of normal liver cells (*P* < 0.05) (Fig. 3B, B1, C, C1, C2). The expression levels of SDF1/CXCR4 in F cells were all higher than those in P cells in vitro and in vivo. The SDF1 levels in F cells were 1.15 times (cDNA, in vitro), 1.57 times (mRNA, in vitro*)* and 1.35 times (mRNA, in vivo*)* higher than those in P cells (*P* < 0.05). The CXCR4 levels in F cells were 3.59 times (cDNA, in vitro), 2.83 times (mRNA, in vitro) and 2.50 times (mRNA, in vivo) higher than those in P cells in vitro and in vivo (P < 0.05) (Fig. 3A, A1, B, B1). When comparing SDF1 and CXCR4, the expression of SDF1 was higher than that of CXCR4 in P cells and normal liver cells, while the expression of CXCR4 was higher than that of SDF1 in F cells in vitro and in vivo. Moreover, the expression difference of CXCR4 between F cells and normal liver cells was much larger than that of SDF1 between F cells and normal liver cells, respectively, both in vitro and in vivo (Figs. 3 and 4).

### The effects of downregulation and upregulation of AnnexinA7 on the expression of SDF1/CXCR4 in hepatocarcinoma cells and transplanted tumor tissues in vitro *and* in vivo

**Fig. 5:**
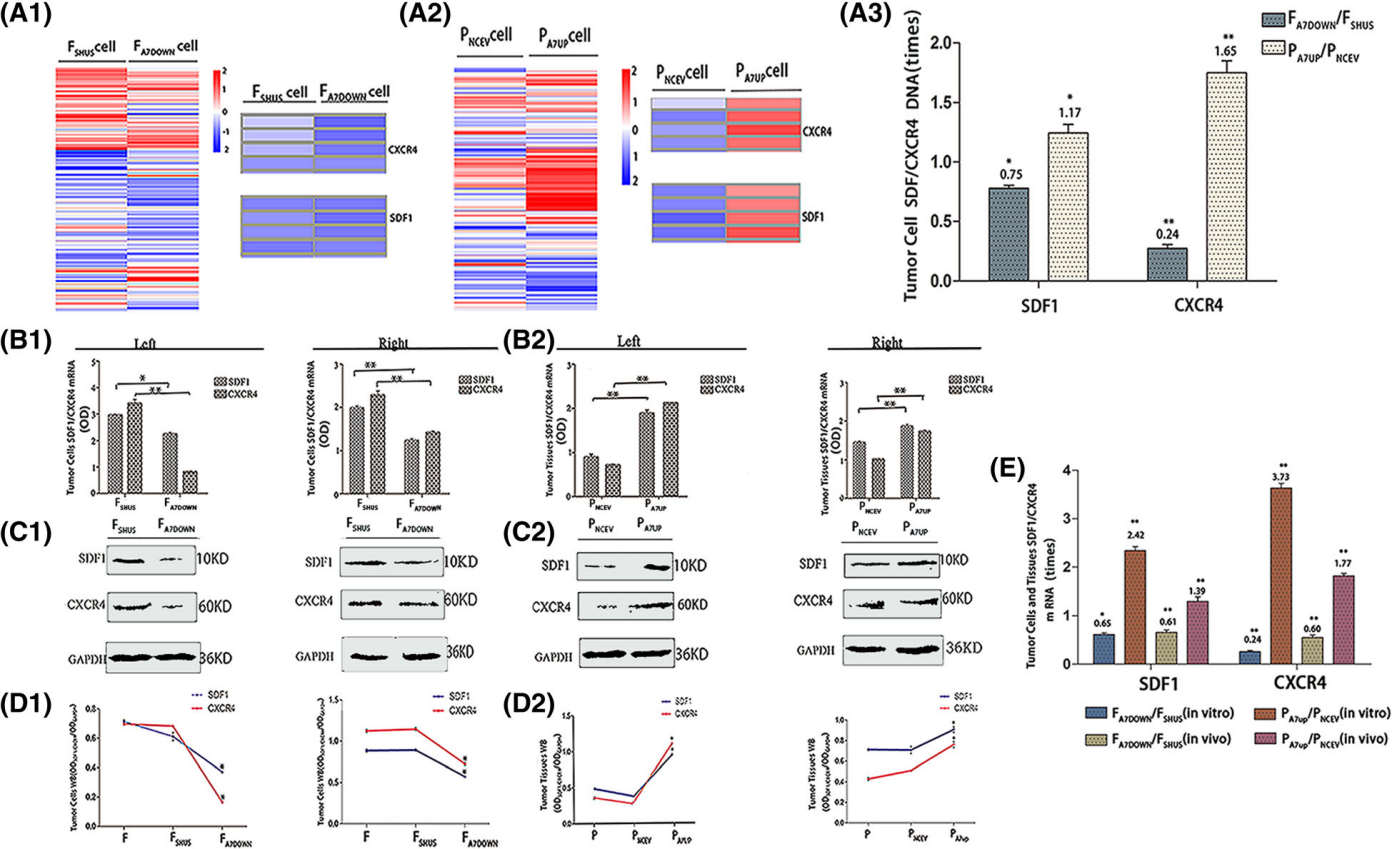
The effects of downregulation and upregulation of AnnexinA7 on the expression levels of SDF1/CXCR4 in hepatocarcinoma cells in vitro and in vivo*.* Transcriptome sequencing heat maps (A1, A2) and cDNA expression (A3) of SDF1/CXCR4 in F_A7DOWN_/F_SHUS_ and P_A7UP_/P_NCEV_ cells. qRT-PCR (B1, B2) and Western blot (C1, C2) analysis of SDF1/CXCR4 mRNA and protein expression respectively, in F_A7DOWN_, F_SHUS_, P_A7UP_, and P_NCEV_ cells in vitro (Left) and in vivo (Right)*.* mRNA expression in F_A7DOWN_ and F_SHUS_ cells (B1, Left) as well as in P_A7UP_/P_NCEV_ cells (B2, Left) in vitro. mRNA expressions in F_A7DOWN_ and F_SHUS_ cells (B1, Right) as well as in P_A7UP_/P_NCEV_ cells (B2, right) in vivo. Protein expressions in F_A7DOWN_ and F_SHUS_ cells (C1, Left) as well as in P_A7UP_, P_NCEV_ cells (C2, Left) in vitro. Protein expressions in F_A7DOWN_ and F_SHUS_ cells (C1, Right) as well as in P_A7UP_ and P_NCEV_ cells (C2, Right) in vivo. mRNA results between different groups in vitro and in vivo (E). Western blot OD densities of SDF1/CXCR4 in F_SHUS_ and F_A7DOWN_ cells (D1, Left) as well as in P_A7UP_ and P_NCEV_ cells *(*D2, Left) in vitro. Western blot OD densities of SDF1/CXCR4 in F_SHUS_ and F_A7DOWN_ cells (D1, Right) as well as in P_A7UP_ and P_NCEV_ cells *(*D2, Right) in vivo

**Fig. 6:**
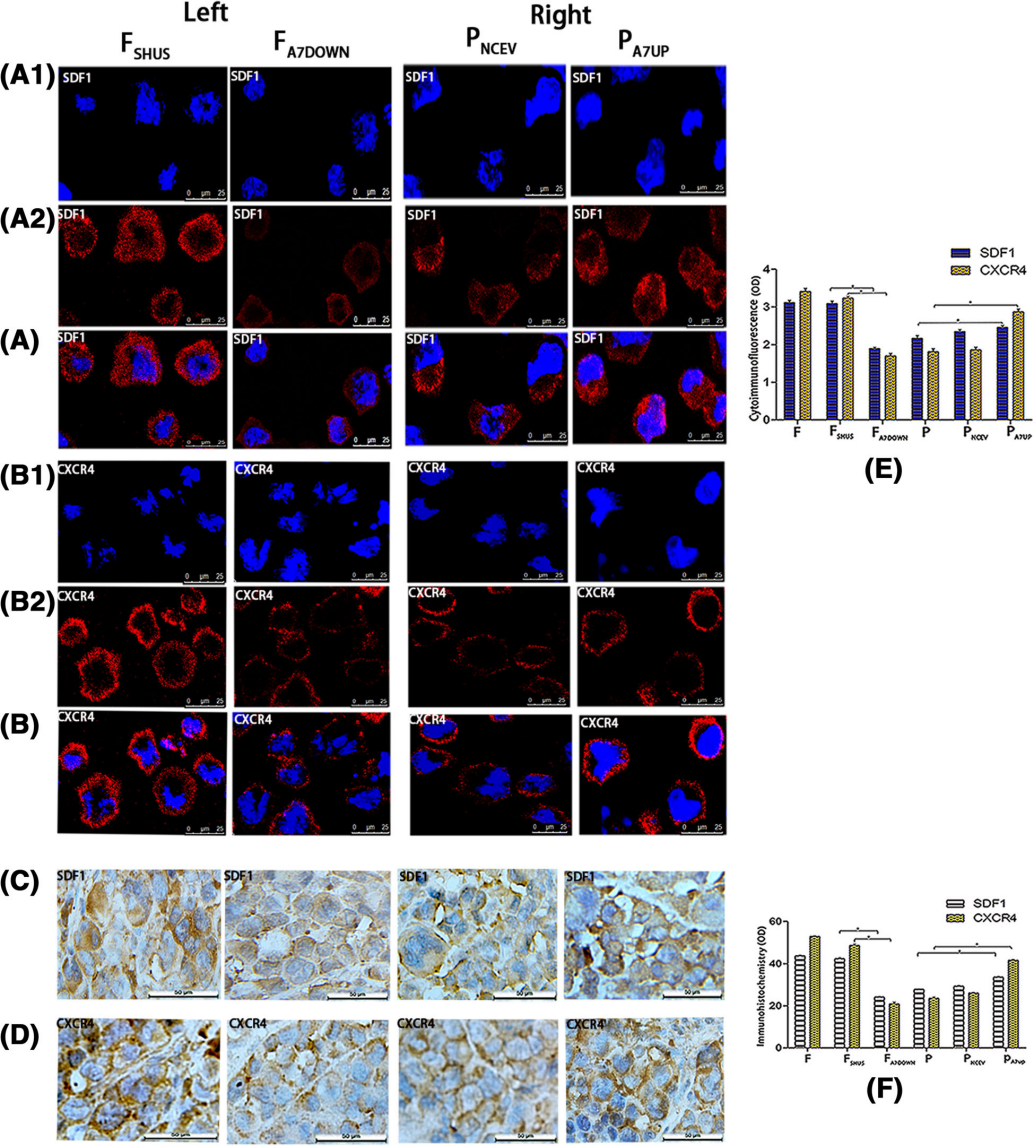
The effects of downregulation and upregulation of AnnexinA7 on the expressions of SDF1/CXCR4 in hepatocarcinoma cells in vitro and in vivo. Cytoimmunofluorescence (cell nuclear DAPI staining A1, B1; cell staining A2, B2; merged picture, A, B) and immunohistochemistry (C, D) analysis of SDF1/CXCR4 expressions in F_SHUS_, F_A7DOWN_ (left), P_NCEV_ and P_A7UP_ (Right) cells in vitro (A1,A2, B1,B2,A, B) and in vivo (C, D). SDF1 expressions in F_SHUS_ and F_A7DOWN_ cells in vitro (A1, A2, A, Left). SDF1 expressions in P_NCEV_ and P_A7UP_ cells in vitro (A1, A2, A, Right). CXCR4 expressions in F_SHUS_ and F_A7DOWN_ cells in vitro (B1, B2, B, Left). CXCR4 expressions in P_NCE and_ P_A7UP_ cells in vitro (B1, B2, B, Right). SDF1 expressions in F_SHUS_ and F_A7DOWN_ cells in vivo (C, Left). SDF1 expressions in P_NCEV_ and P_A7UP_ cells in vivo (C, Right). CXCR4 expressions in F_SHUS_ and F_A7DOWN_ cells in vivo (D, Left). CXCR4 expressions in P_NCEV_ and P_A7UP_ cells in vivo (D, Right). Cytoimmunofluorescence and immunohistochemistry results (OD) for SDF1/CXCR4 in F_SHUS_, F_A7DOWN_, P_NCEV_ and P_A7UP_ cells in vitro (E)and in vivo (F); _*_ indicates that P < 0.05 as statistically significant

Transcriptome sequencing, qRT-PCR, Western blotting, cytoimmunofluorescence and immunohistochemistry showed that SDF1 was mainly localized in the cytoplasm of cells, and in a small amount, it was located in the cell membrane; while CXCR4 was mainly localized in the cell membrane, and in a small amount, it was localized in the cytoplasm in F/P, F_A7DOWN_/P_A7UP_ and F_SHUS_/P_NCEV_ cells both in vitro and in vivo (Fig. 6A, B, C, D). More importantly, there was a significant positive relationship between the expression of SDF1/CXCR4 and AnnexinA7 gene regulation. The downregulation or upregulation of AnnexinA7 resulted in decreased or increased expression of SDF1/CXCR4, respectively, showing a highly homotropic pattern. After downregulation of AnnexinA7, the expression levels of SDF1/CXCR4 in F_A7DOWN_ cells were lower than those in F_SHUS_ and F cells in vitro and in vivo. The SDF1 level of F_A7DOWN_ cells was decreased by 24.76% (cDNA, in vitro) and 34.17% (protein, in vitro) compared to that in F_SHUS_ cells (*P* < 0.05), and the CXCR4 level in F_A7DOWN_ cells was decreased by 75.68% (cDNA, in vitro) and 75.38% (protein, in vitro) compared to that in F_SHUS_ cells (*P* < 0.05) (Fig. 5A3, B1, C1, D1, E). After upregulation of AnnexinA7, the expression levels of SDF1/CXCR4 in P_A7UP_ cells were higher than those in P_NCEV_ cells in vitro and in vivo. After AnnexinA7 was upregulated, the SDF1 level in P_A7UP_ cells was 1.17 times (cDNA, in vitro) and 2.42 times (mRNA, in vivo) higher than that in P_NCEV_ cells, and the CXCR4 level in P_A7UP_ cells was 1.65 times (cDNA, in vitro) and 3.73 times (mRNA, in vivo) higher than that in P_NCEV_ cells (P < 0.05) (Fig. 5 A3, B2, C2, D2, E). Similarly, cytoimmunofluorescence and immunohistochemistry also showed that the fluorescence and staining intensities of CXCR4/SDF1 were all lower in F_A7DOWN_ cells and higher in P_A7UP_ cells compared to respective control groups of F_SHUS_/P_NCEV_ cells in vitro and in vivo (Fig. 6). Moreover, compared to the expression levels of the SDF1 ligand, the expression level of the CXCR4 receptor were modulated more by downregulation and upregulation of AnnexinA7 (Figs. 5 and 6). After downregulation or upregulation of AnnexinA7, the SDF1 expression levels in F_A7DOWN_ and P_A7UP_ cells were 34.17% *(*protein, in vitro*)* lower or 2.39 times (protein, in vitro) higher than those in F_SHUS and_ P_NCEV_ cells (*P* < 0.05) respectively, while the CXCR4 expression levels in F_A7DOWN_ and P_A7UP_ cells were 75.83% (protein, in vitro) lower or 3.24 times (protein, in vitro) higher than those in F_SHUS and_ P_NCEV_ cells, respectively (P < 0.05) (Fig. 5C1, C2, D1, D2). In addition, the expression differences of CXCR4 between F_A7DOWN_/P_A7UP_ cells and F_SHUS_/P_NCEV_ cells were much larger than those of SDF1 between F_A7DOWN_/P_A7UP_ cells and F_SHUS_/P_NCEV_ cells, respectively, in vitro and in vivo (Figs. 5 and 6).

## Discussion

SDF1 is a small secretory molecule and is one of the chemokine family members, and it is also known as CXCL12 [7]. The human SDF1 gene is located in chromosome 10q11.1, which codes 89 and 93 amino acid polypeptides. SDF1 has two isomers, namely, SDF1α and SDF1β. Moreover, the expression, regulation and function of the two isomers are similar. The human CXCR4 gene is located at chromosome 2q22.1, and it is a G protein-coupled receptor composed of 352 amino acids. CXCR4 is the only specific receptor for SDF1, and SDF1 binds to the N-side of CXCR4 and interacts with the second extracellular loop (ECL2) of CXCR4 [8, 9]. SDF1 and CXCR4 are expressed in a variety of normal cells and tissues, including lymphocytes and macrophages as well as brain, heart, kidney, liver, lung and spleen. The binding of SDF1 with CXCR4 activates molecular networks of downstream signaling pathways, promoting cell proliferation and movement, inducing immune tolerance and inflammation, affecting migration and homing of hematopoietic stem cells, and stimulating embryonic development, especially malignant tumor growth and progression [10–12].

Existing data indicate that the SDF1 ligand and the CXCR4 receptor are expressed in different types of malignant tumor cells [13, 14]. It has been shown that CXCR4 is increased by 2–15 times in ovarian cancer cells compared to normal ovarian cells at the mRNA level [15, 16]. Some studies have found that SDF1 and CXCR4 are highly expressed in cholangiocarcinoma [17], breast cancer [18], non-small cell lung cancer [19, 20], cervical cancer [21, 22], liver cancer Huh7 cells [23] and melanoma cells [24]. Hypoxia also induces the expression of SDF1/CXCR4 in tumor cells [25, 26]. In mouse breast cancer models, inhibitors of CXCR4 significantly reduce the number of breast cancer metastatic cells to lymph nodes [27]. Studies on colon cancers and liver cancers have suggested that SDF1 and CXCR4 are involved in cancer lymphatic metastasis and that the mechanism is related to the induction of cell proliferation and directional migration [28, 29]. The expression of SDF1 is positively correlated with CXCR4 in epithelial ovarian cancer, in which both SDF1/CXCR4 are involved in cell malignant transformation and take part in the development, invasion and metastasis of ovarian malignancies [30].

The Hca-F and Hca-P (F and P) cell lines are subclones derived from the same parent cells of mouse hepatocarcinoma ascitic cells by our laboratory many years ago. In tumor-bearing mice, Hca-F cells have an approximate 70% lymph node metastasis rate and Hca-P cells have an approximate 30% lymph node metastasis. Therefore, the two strains are good models for researching the molecular mechanism of different lymphatic metastatic potentials of mouse hepatocarcinoma cells [31–33]. AnnexinA7 is a protein that is correlated with membrane transport, signal transduction, proliferation, migration and other biological behaviors of tumors, including mouse hepatocarcinoma. Our previous studies have found that the increased or reduced expression levels of AnnexinA7 gene promote or inhibit the proliferation, migration and invasion of Hca-F and Hca-P tumor cells in vitro [4, 31, 34]. It has also been reported by our laboratory that several other genes and proteins in mouse liver cancer cells are positively regulated by AnnexinA7 modulation, such as Galectin-3, GSN, Rack1, Sorcin, Plectin, SODD, HnRNPA2/B1 and RBM, or negatively regulated by AnnexinA7 modulation, such as Jnk1,Clic1, Ech1 and Ezrin [3, 4, 33–39]. However, until now, there have been no reports on whether the expression of SDF1/CXCR4 is positively or negatively regulated by AnnexinA7.

In this paper, the results of gene transcriptome sequencing, qTR-PCR, Western blotting, cytoimmunofluorescence and immunohistochemistry show that both SDF1 and CXCR4 are present in different tumor cells and even normal liver cells in vitro and in vivo before and after AnnexinA7 regulation. Furthermore, other researchers have provided several evidences of SDF1/CXCR4 co-expression in breast cancer, colorectal cancer and glioma [40–42]. Co-expression of SDF1/CXCR4 molecules, either before or after AnnexinA7 regulation, indicates that they can exist alone in different cells but commonly exist in the same cell and may play a biological role through paracrine and autocrine mechanisms. Higher expression levels of SDF1/CXCR4 in hepatocarcinoma cells are found compared to normal liver cells in vitro and in vivo*,* which favor cell proliferation and metastasis of tumor cells. Furthermore, the expression levels of SDF1/CXCR4 in F cells with high lymphatic metastatic potential are higher than those in P cells with low lymphatic metastatic potential in vitro and in vivo*.* The expression levels of SDF1/CXCR4 decrease or increase in synchrony following the downregulation or upregulation of the AnnexinA7 gene. These results not only indicate that the downregulation or upregulation of the AnnexinA7 gene can induce synchronous inhibition or promotion of SDF1/CXCR4 expressions but also indicate that SDF1/CXCR4 are the functional downstream molecules of AnnexinA7. To the best of our knowledge, the current data show for the first time that AnnexinA7 regulates SDF1/CXCR4 presentation in target cells, yet the regulatory mechanism of AnnexinA7 to SDF1/CXCR4 axis heretofore has not been understood. It is necessary to further clarify its mechanism based on interesting findings of this article. However, some relative reports have been presented recently. For example, some authors found out that Annexin family members, AnnexinA1 and AnnexinA2, bound to and immunoprecipitated with SDF1, and Annexin A2 co-localized with SDF1 in the cells. Furthermore, it has been noticed that AnnexinA2 can also bind to formyl peptide receptor2 in mice and humans, which is G protein-coupled receptor, like CXCR4 in cell membrane, and may interact with chemotactic factors, like SDF1 [43–45].

Compared to SDF1 expression levels, the expression levels of CXCR4 in F cells and corresponding transplanted tumor tissues were increased to a greater extent than that in normal liver cells, P cells and normal liver tissue as well as corresponding transplanted tumors. Moreover, compared to SDF1 after AnnexinA7 downregulation or upregulation, the expression levels of CXCR4 in F_A7DOWN_/P_A7UP_ cells decreased or increased to a greater extent than that in F_SHUS_/P_NCEV_ cells in vitro and in vivo with sharp increasing or decreasing slopes (shown in Fig. 5 A3, D1, D2, E; Fig. 6E, F). Therefore, these data further suggest that strong expression of CXCR4 is more important for a cancer phenotype with high lymphatic metastatic potential than SDF1. CXCR4, and not SDF-1, is more sensitive to AnnexinA7 downregulation and upregulation. Thus, CXCR4 is more likely to be a good indicator for tumor malignant behaviors. As a cell membrane structural protein, CXCR4 is more susceptible to the regulation of intracellular gene networks compared to SDF1 as a cell secreting molecule. As a result, CXCR4 expression more directly reflects the biological characteristics of cancer cells. This distinction in sensitivity and efficiency between SDF1 and CXCR4, after AnnexinA7 down and up regulations, has probably come from that both AnnexinA7 and CXCR4 are transmembrane protein molecules located on the same cell membrane, which are closer to each other geographically. Meanwhile they are calcium-dependent phospholipid binding protein or calcium-mediated transduction signalling protein, sharing the same molecular mechanism like activation for intracellular calcium.

The relationship between VEGFC/D-VEGFR3/NRP2 and SDF1/CXCR4 is important not only because these two pairs of ligand-receptor have the same function to promote cell proliferation but also because they are often consistent and highly expressed in certain cells. For example, CXCR4 is involved in the process of lymphatic endothelial cell generation and nerve cell orientation, consistent with the main functions of VEGFR3 and NRP2, respectively [8, 46–48]. The latest studies have found that VEGFC specifically stimulates the expression of CXCR4 in lymphangiogenic endothelial cells and that VEGFD promotes breast cancer and cervical cancer cell adhesion, migration and metastasis through CXCR4 binding signals, while they have also shown that CXCR4 induces the expression of VEGFD through a feedback loop, indicating that the VEGFC/D/VEGFR3 axis and SDF1/CXCR4 axis might have several superimposed overlapping effects [22, 49]. The expression changes of VEGFC/D-VEGFR3/NRP2 and SDF1/CXCR4 are almost completely identical between tumor cells and normal cells, between high and low lymph node metastatic potential of liver cancer cells as well as between downregulation and upregulation of AnnexinA7 (unpublished data). Therefore, it is reasonable to suppose that AnnexinA7 through the coordination of VEGFC/D-VEGFR3/NRP2, and SDF1/CXCR4 may affect the process of tumor development and progression. Thus, SDF1/CXCR4 should be considered as a pair of VEGFC/D-VEGFR3/NRP2 axis-related downstream molecules.

This study mainly examines the expression differences of the SDF1 ligand and the CXCR4 receptor in mouse hepatocarcinoma cells with different lymphatic metastasis abilities and in mouse normal liver cells in vitro and in vivo*.* This study reveals the effects of AnnexinA7 downregulation and upregulation on the SDF1/CXCR4 axis in mouse liver cancer cells in vitro and in vivo. According to previous reports and the results of our studies, the mechanisms of SDF1/CXCR4 in tumor development and progression may be as follows: to stimulate tumor cell autocrine and paracrine growth signaling factors, provoking cell proliferation and anti-apoptosis; to cooperate with other molecular networks, strengthening the activity of related pathways; to adjust the synthesis and degradation of actins and extracellular matrix, causing tumor cell movement and invasion; and to regulate the activity of adhesion molecules on tumor cell surfaces, enhancing the recognition, chemotaxis and adhesion to endothelial cells. However, it remains unknown how the generation and secretion of SDF1/CXCR4 molecules correlate in the same cancer cell and how they interact with upstream and downstream pathways, e.g., AnnexinA7 and VEGFC/D-VEGFR3/NRP2 during tumor development and progression. Thus, further studies are required.

## Conclusions

Our experiments show that the co-expression levels of SDF1/CXCR4 in F/P cells in vitro and corresponding tumor tissues in vivo are higher than those in normal liver cells and normal liver tissue at cDNA, mRNA and protein levels as well as cytoimmunofluorescence and immunohistochemistry intensities. Moreover, the levels are also higher in F cells with high lymphatic metastatic potential and corresponding tumor tissue compared to those in P cells with low lymphatic metastatic potential and corresponding tumor tissue. With downregulation or upregulation of AnnexinA7, the expression levels of SDF1/CXCR4 are uniformly decreased or increased in cancer cells in vitro and in vivo. Moreover, the expression levels of CXCR4 are influenced more by the regulation of AnnexinA7 than those of SDF1. Furthermore, the co-expression of SDF1 and CXCR4 is a molecular characteristic of hepatocarcinoma cells, especially those with high lymphatic metastatic potential. AnnexinA7 positively regulates the expression of SDF1/CXCR4, especially CXCR4, and AnnexinA7 is a functional regulator of the SDF1/CXCR4 axis, which influences the occurrence and development of tumors.

## Abbreviations

A7: AnnexinA7
CIF: cytoimmunofluorescence
CXCR4: chemokine CXC receptor 4
F_A7DOWN_: lentivirus stable transfected AnnexinA7 downregulated HcaF cells
F_SHUS_: transfected AnnexinA7 unrelated sequence HcaF cells
IHC: immunohistochemistry
NRP2: Neuropilin2
P_A7UP_: AnnexinA7 overexpression HcaP cells
P_NCEV_: transfected empty vector HcaP cells
SDF1: stromal cell-derived factor1
VEGFC/D: vascular endothelial growth factor-C/D
VEGFR3: vascular endothelial growth factor receptor 3
